# A Japanese registry study and systematic review of particle therapy for renal cell carcinoma

**DOI:** 10.1093/jrr/rrad010

**Published:** 2023-04-12

**Authors:** Hitoshi Ishikawa, Takeshi Arimura, Kazushi Maruo, Hidemasa Kawamura, Shingo Toyama, Takashi Ogino, Tomoaki Okimoto, Masao Murakami, Yoshitaka Sato, Kentaro Nishioka, Masayuki Araya, Hisateru Ohba, Kensuke Umehara, Hidefumi Aoyama, Wataru Obara, Haruhito Azuma, Hiroshi Tsuji, Hideyuki Sakurai

**Affiliations:** QST Hospital, National Institutes for Quantum Science and Technology, Inage, Chiba 263-8555, Japan; Department of Radiation Oncology, University of Tsukuba, Faculty of Medicine, Tsukuba, Ibaraki 305-8575, Japan; Medipolis Proton Therapy and Research Center, Ibusuki, Kagoshima 891-0304, Japan; Department of Biostatistics, University of Tsukuba, Faculty of Medicine, Tsukuba, Ibaraki 305-8575, Japan; Gunma University Heavy Ion Medical Center, Maebashi, Gunma 371-8511, Japan; Ion Beam Therapy Center, SAGA-HIMAT Foundation, Tosu, Saga 841-0071, Japan; Medipolis Proton Therapy and Research Center, Ibusuki, Kagoshima 891-0304, Japan; Department of Radiology, Hyogo Ion Beam Medical Center, Tatsuno, Hyogo 679-5165, Japan; Department of Radiation Oncology, Southern Tohoku Proton Therapy Center, Koriyama, Fukushima 963-8052 Japan; Proton Therapy Center, Fukui Prefectural Hospital, Fukui, Fukui 910-8526, Japan; Global Center for Biomedical Science and Engineering, Hokkaido University Faculty of Medicine, Hokkaido 060-8638, Japan; Proton Therapy Centre, Aizawa Hospital, Matsumoto, Nagano 390-8510, Japan; QST Hospital, National Institutes for Quantum Science and Technology, Inage, Chiba 263-8555, Japan; QST Hospital, National Institutes for Quantum Science and Technology, Inage, Chiba 263-8555, Japan; Department of Radiation Oncology, Hokkaido University Faculty of Medicine, Hokkaido 060-8638, Japan; Department of Urology, Iwate Medical University, School of Medicine, Yahaba-Cho, Iwate 028-3694, Japan; Department of Urology, Osaka Medical and Pharmaceutical University, Takatsuki, Osaka 569-8686, Japan; QST Hospital, National Institutes for Quantum Science and Technology, Inage, Chiba 263-8555, Japan; Department of Radiation Oncology, University of Tsukuba, Faculty of Medicine, Tsukuba, Ibaraki 305-8575, Japan

**Keywords:** renal cell carcinoma (RCC), particle beam therapy (PBT), proton beam therapy, carbon-ion radiotherapy (CIRT), survival, toxicity

## Abstract

The feasibility and efficacy of particle beam therapy (PBT) using protons or carbon ions were compared with those of photon-based stereotactic body radiotherapy (SBRT) for primary renal cell carcinoma (RCC) via a systematic review and nationwide registry for PBT (Japanese Society for Radiation Oncology [JASTRO] particle therapy committee). Between July 2016 and May 2019, 20 patients with non-metastatic RCC who were treated at six Japanese institutes (using protons at three, using carbon ions at the other three) were registered in the nationwide database and followed up prospectively. The 20 patients comprised 15 men and had a median age of 67 (range: 57–88) years. The total radiation dose was 66–79.6 Gy (relative biological effectiveness [RBE]). Over a median follow up of 31 months, the 3-year rates of overall survival (OS) and local control (LC) were 100% and 94.4%, respectively. No grade ≥ 3 toxicities were observed. Based on a random effects model, a meta-analysis including the present results revealed 3-year OS rates after SBRT and PBT of 75.3% (95% CI: 57.3–86.6) and 94.3% (95% CI: 86.8–97.6), respectively (*P* = 0.005), but the difference in LC rates between the two methods was not observed (*P* = 0.63). PBT is expected to have similar if not better treatment results compared with SBRT for primary renal cancer. In particular, PBT was shown to be effective even for large RCC and could provide a therapeutic option when SBRT is not indicated.

## INTRODUCTION

The Japanese Cancer Registry estimated that approximately 30 000 new cases of renal cancers were diagnosed in 2019 [1], and renal cell carcinoma (RCC) accounts for most of these cases. The gold standard treatment for patients with primary RCC is surgery, to remove either the entire kidney and surrounding tissues (radical nephrectomy) or the tumor (partial nephrectomy) [2–4]. Partial nephrectomy is a nephron-sparing surgery appropriate for localized small-sized tumors where technically feasible [5]. Radiofrequency ablation and cryoablation are alternative treatments for patients who do not meet the criteria for surgery, but these approaches are invasive and limited to small tumors located on the dorsal side of the kidney and distant from vascular structures and the upper urinary tract [6, 7]. RCC predominantly affects the older population, and these ablative therapies sometimes cause issues in patients requiring continuous anticoagulative medication.

As an alternative to ablative treatments, radiotherapy (RT) is a non-surgical and non-invasive treatment option for RCCs. However, normal tissues, including the normal kidney and especially the gastrointestinal (GI) tract, are radiosensitive. RCC is considered resistant to radiation delivered using conventional fractionation schedules (1.8–2 Gy per fraction) and requires high radiation doses to eradicate the cancer cells [8]. Stereotactic body radiotherapy (SBRT) is an emerging noninvasive treatment delivered in a few treatment sessions at a large fractional dose, and some studies have shown the efficacy of SBRT for RCC, with low toxicity and excellent LC rates [9–11]. However, no randomized trials have compared SBRT with radiofrequency ablation or cryoablation for small-sized RCCs, and there is insufficient evidence to recommend a standard dose fractionation or technique.

The efficacy of hypofractionated particle beam therapy (PBT) for RCC has been shown in a small number of studies. The physical properties of protons and carbon ions allow for better dose distributions compared with photon-based RT. Since the area of normal tissues surrounding the kidney irradiated with a low-to-intermediate dose of SBRT will increase with tumor diameter, it is anticipated that hypofractionated PBT can control radioresistant RCC tumors without increasing the rate of late adverse effects such as GI bleeding and chronic renal dysfunction. At the National Institutes of Quantum Science and Technology, the feasibility and effectiveness of hypofractionated carbon-ion RT (CIRT) for RCC have been confirmed in phase I/II studies, and the number of treatment sessions has been reduced from 16 fractions over 4 weeks to 4 fractions over 1–2 weeks, step by step [8]. Proton beam therapy, another type of PBT, was evaluated by a multi-institutional retrospective study involving 22 RCC patients treated at six Japanese institutes [12]. In that study, the 3-year overall survival (OS) and disease-specific survival rates were 95% and 100%, respectively, over a median follow up of 37 months and neither recurrence nor grade 3 adverse events were observed. However, no further studies of proton beam therapy for RCC have been published.

The Japanese Society for Radiation Oncology (JASTRO) has created a registry of all patients under advanced medical treatment in Japan with the aim of establishing PBT coverage by the national health insurance system in Japan. Although PBT for several cancers, such as prostate cancer and bone and soft tissue sarcoma, is already covered by the insurance system [13], PBT for many other types of cancers including RCC is still performed as advanced medical treatment. The JASTRO working group continues to investigate the prospectively collected registry data. Our aims of the present study are to confirm whether treatment outcomes obtained from the prospective registry can reproduce results of the previous PBT studies and to also compare the outcomes of PBT with those of SBRT for RCC by a meta-analysis based on a systematic review and registry data analysis.

## MATERIALS AND METHODS

### Analysis of the registry data for PBT

The Japanese multi-institutional registry has been conducted since May 2016, and the data collected from RCC patients who received proton therapy or CIRT between May 2016 and June 2018 were investigated to evaluate the rates of OS, progression-free survival (PFS), local control (LC) and treatment-related late GI toxicities of grade ≥ 3. Patients with primary histopathologically or clinically diagnosed RCC without metastases at any site were eligible for PBT as advanced medical treatment in Japan. However, patients not expected to survive long-term after PBT due to severe comorbidities and/or other cancers were ineligible. The registry data analyses were approved by the institutional review boards and the studies were registered onto the University Hospital Medical Information Network (UMIN) Center (UMIN000022917 for proton therapy and UMIN000024709) for CIRT. A written consent was obtained from each patient.

The follow-up period was defined from the first day of PBT to the date of death or the last follow-up visit. Generally, patients were followed at 3-month intervals during the first year after PBT and at 6-month intervals thereafter. OS was defined as the time from the initiation of PBT to death irrespective of the reason, and PFS was defined as the time from the initiation of PBT to death or any sign of cancer progression. The response of the tumor to PBT was classified as a complete response, partial response, stable disease, or progressive disease according to the Response Evaluation Criteria in Solid Tumors, version 1.1 [14]. LC was defined as the time from the initiation of PBT to any sign of progression of the treated lesions. The OS, PFS and LC rates were calculated using the Kaplan–Meier method for the period from PBT initiation to the date of the event or last follow-up visit. Treatment-related late GI toxicities of grade ≥ 3, which were defined as complications occurring 3 months after the end of PBT, were evaluated according to the Common Terminology Criteria for Adverse Events (version 4.0) [15].

### Systematic review of SBRT and PBT

The systematic review was performed in accordance with the Preferred Reporting Items for Systematic Review and Meta-Analyses (PRISMA) guidelines [16]. The library at the National Institutes for Quantum Science and Technology conducted a systematic search for articles on RT for RCC that were published in English between January 2000 and September 2020. The search terms were as follows: (‘renal cancer*’[TIAB] OR ‘renal cell carcinoma*’[TIAB] OR ‘kidney’[MH]) OR (‘cancer’[TIAB] ‘cancers’[TIAB] OR ‘neoplasm’ [TIAB] ‘neoplasms’[TIAB] OR ‘carcinomas’[TIAB] OR ‘tumor’ [TIAB] OR ‘tumors’[TIAB] OR ‘tumour’[TIAB] OR ‘carcinoma’ [TIAB] OR ‘carcinomas’[TIAB]) AND (‘X-irradiation’[TIAB] OR ‘X-ray irradiation’[TIAB] OR ‘X-radiation’[TIAB] OR ‘X-ray radiation’[TIAB] OR ‘X-ray therapy’[TIAB] OR ‘X-ray treatment’ [TIAB] OR ‘X-rays’[MH] OR ‘X-ray therapy’[MH] OR ‘BT’[TIAB] OR ‘IMRT’[TIAB] OR ‘3D-CRT’[TIAB] OR ‘SRT’[TIAB] OR ‘SBRT’[TIAB] OR ‘radiation therapy’[TIAB] OR ‘radiotherapy’ [TIAB]) AND (‘primary’[TIAB] NOT ‘metasta*’[TIAB] OR ‘neoplasm metastasis’[MH] OR ‘oligometa*’[TIAB] OR ‘oligo-recurren*’[TIAB]). The inclusion criteria for the literature search were defined using the Population, Intervention, Control and Outcome approach. Because of the limited number of articles found on RT or PBT for primary RCC, clinical trials (retrospective as well as prospective) that included at least six patients and reported at least one of the following outcomes were eligible: rates of OS, PFS, LC and grade 3 GI toxicities. However, case studies, abstracts, preclinical studies and review papers were excluded. When multiple publications were reported from a single institution, outcome data from only the most recent or most relevant study were included.

A manual search of articles/papers based on information provided by the systematic review team (H.I., T.A., H.K., S. T. and T.O.) was also conducted. In the first screening, two radiation oncologists independently reviewed the retrieved articles and selected potentially relevant articles based on the titles and abstracts. In the second screening, the full text of the studies was reviewed by another reviewer in addition to the two radiation oncologists to select the studies meeting our criteria. Any disagreement was resolved by mutual discussion.

### Meta-analysis of the systematic review and registry data to compare SBRT and PBT

For the Kaplan–Meier estimates of the 3-year OS, PFS and LC rates for each treatment, log–log transformed estimates were generated by the random effects model based on the restricted maximum likelihood method and were then back-transformed. The I^2^ statistic was estimated in each meta-analysis.

If articles did not report these 3-year rates, the rates were estimated directly from the survival curves. If precision information (confidence interval [CI]) was missing, it was imputed using the number of subjects, risk set size at 3 years and dropout rate. Assuming that the background factors were sufficiently similar between the treatments during the study selection process, we compared the meta-analysis results between the treatments using the Wald-type test. The significance level of the statistical tests was set at 0.05, and 95% CIs were used. The statistical analyses were conducted using R software (R Core Team, Vienna, Austria) and meta package in R software [17].

## RESULTS

### Analysis of the Japanese multi-institutional registry data set

Between May 2016 and June 2018, 20 primary RCC patients (15 males and five females) were treated with proton beam therapy (*n* = 16) or CIRT (*n* = 4). All patients completed the scheduled treatments successfully and were eligible for the present study. The patient characteristics are shown in Table 1.

**Table 1:** Characteristics of the patients with renal cell carcinoma from the registry data set

Age (years)	Median: 67 (range: 57–88)
< 69	13 (65%)
≥ 70	7 (35%)
Sex	
Male	15 (75%)
Female	5 (25%)
T stage	
T1a	15 (75%)
T1b	3 (15%)
T2	1 (5%)
T3	1 (5%)
Tumor size (mm)	Median: 35 (range: 13–73)
< 40	15 (75%)
≥ 40	5 (25%)
Tumor location	
Inner/ventral area	15 (75%)
Outer/dorsal area	5 (25%)
Treatment modality	
Proton beam therapy	16 (80%)
Carbon-ion radiotherapy	4 (20%)
Dose fractionation (Gy(RBE)/fr)	
Proton beam therapy	
66.0/10 (BED_5_: 153 Gy)	5 (25%)
76.0/20 (BED_5_: 133.8 Gy)	8 (40%)
77.0/35 (BED_5_: 110.9 Gy)	3 (15%)
Carbon-ion radiotherapy	
66.0/12 (BED_5_: 138.6 Gy)	3 (15%)
72.0/12 (BED_5_: 158.4 Gy)	1 (5%)

Gy(RBE)/fr: gray (relative biological effectiveness)/fraction

The median age of the patients was 67 (range, 57–88) years. Among the patients, three were medically inoperable, whereas the remaining 17 patients refused to receive surgery. The median diameter of the 20 tumors was 3 (range, 1.3–7.3) cm, and there were two non-T1 tumors. Fifteen (75%) tumors were located at the inner/ventral side of the kidney, indicating planning target volumes (PTVs) were close to GI tracts and the remaining five (15%) tumors existed in the outer/dorsal areas of the kidney. The dose fractionation schedules for proton beam therapy and CIRT are also shown in Table 1. The median biological effective doses (BEDs) obtained using the linear-quadratic model with α/β = 3 (BED_3_) for the normal kidney and GI tract and α/β = 5 (BED_5_) for RCC were 172.3 (range, 133.5–216.0) Gy (relative biological effectiveness [RBE]) and 133.8 (range, 110.9–158.4) Gy (RBE) [18, 19], when the RBEs of proton and carbon-ion beams were set to 1.1 and 3.0, respectively [12, 13, 20].

**Fig. 1:**
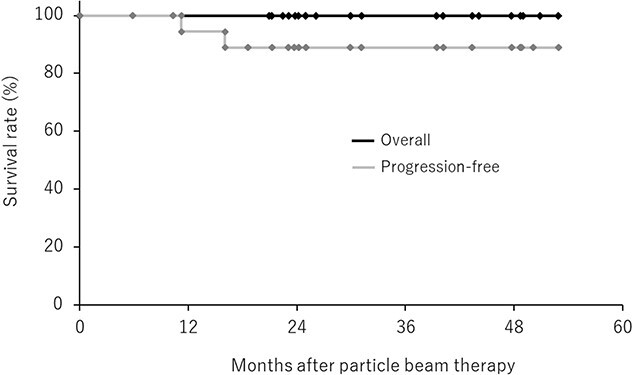
OS and PFS curves for RCC patients after PBT.

The median follow-up period was 31 (range, 21–52.9) months. Of the 20 patients, one (5%) had local progression, and the 3-year LC rate was 94.4% (95% CI, 83.8–100%). Distant metastasis developed in another patient, but all patients were still alive at the last follow up. The 3-year OS and PFS rates were 100% and 88.9% (95% CI, 74.4–100%), respectively (Fig. 1). Regarding treatment-related adverse effects, no grade 3 GI toxicities were observed. The median reduction in the estimated glomerular filtration rate (eGFR) from the start of PBT to the last follow up was 5.6 ml/min/1.73 m^2^ (0–32.38 ml/min/1.73 m^2^). No patient underwent artificial dialysis after PBT.

### Systematic review

**Fig. 2:**
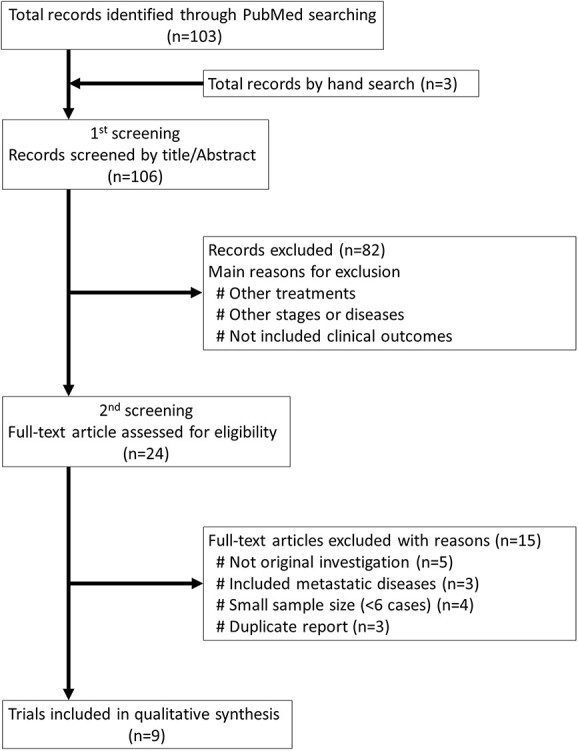
PRISMA flow diagram of the systematic search strategy.

Figure 2 shows the PRISMA flow diagram of the systematic search strategy. The systematic search yielded 103 publications, and an additional three publications were selected by hand search. Of these, 24 studies were evaluated in the second screening, of which a total of nine studies (six using SBRT and three using PBT) were finally selected for the qualitative analysis [12, 21–28]. Table 2 summarizes all of the relevant details of these studies. The three PBT articles included the clinical outcomes after proton beam therapy (*n* = 1) or CIRT (*n* = 2), and all were conducted in Japan. Two Japanese SBRT studies investigated patients with T1 tumors only. The proportion of T2 or higher cases in all SBRT studies ranged from 0 to 4.8%, and the weighted average was 2.3%. In the PBT series, the corresponding proportion ranged from 9.1% to 15.8%, with a weighted average of 14.2%. In addition, the median or mean age of the patients seemed younger in the PBT than SBRT series (Table 2).

**Table 2:** Treatment and patients in studies of SBRT or PBT for renal cell carcinoma

Author (Year)	Reference number	Study type	No. of patients	Proportion of non-T1 tumors (%)	Size (mm)	Age (years)	Dose fractionation (Gy(RBE)/fr)	BED_5_ (Gy)
**SBRT**								
Ponsky (2015)	21	Prospective	19	NA	57.9 cm^3^	77^*^	24–48/4	52.8–163.2
Yamamoto (2016)	22	Prospective	14	0%	30^*^	75^*^	50–70/10	100–168
Siva (2017)	23	Prospective	37	2.7%	48^*^	78^*^	26/1 or 42/3	159.6–164.2
Siva (2018)	24	Retrospective	223	NA	43^¶^	72^¶^	14–26/1, 24/2–70/10	150^†^
Funayama (2019)	25	Prospective	13	0%	19^*^	72^*^	60/10 or 70/10	132–168
Peddada (2020)	26	Prospective	21	4.8%	29^*^	71^*^	42/3 or 48/3	159.6–201.6
**Proton beam therapy**								
Fukumitsu (2020)	12	Retrospective	22	9.1%	35^*^	67^*^	Varying	110.9–153
**CIRT**								
Kasuya (2018)	27	Retrospective	19	15.8%	36^*^	67^*^	64, 72, 80/16 or 66/12	115–160
Kasuya (2019)	28	Prospective	8	12.5%	43^*^	69^*^	66/12 or 72/12	138.6–158.4

*Abbreviations*: SBRT, stereotactic body radiotherapy; CIRT, carbon-ion radiotherapy, BED, biological effective dose

^*^Median

^¶^Mean

^†^representative dose

In the SBRT series, the OS, PFS and LC rates were 72–92%, 77–89% and 92–100% at 2 years and 71–88%, 65–88% and 92–100% at 3–5 years, respectively. The OS, PFS and LC rates at 3–5 years after PBT were 88–95%, 69–95% and 94–100%, respectively. Regarding adverse effects, the incidence of grade ≥ 3 GI toxicities in the SBRT series ranged from 0% to 10.5%, with a weighted average of 1.8%, but toxicity was not observed in the PBT series. In addition, the weighted average reductions in the serum eGFR after SBRT and PBT were 6.7 ml/min/1.73 m^2^ and 7.6 ml/min/1.73 m^2^, respectively.

### Meta-analysis

**Fig. 3:**
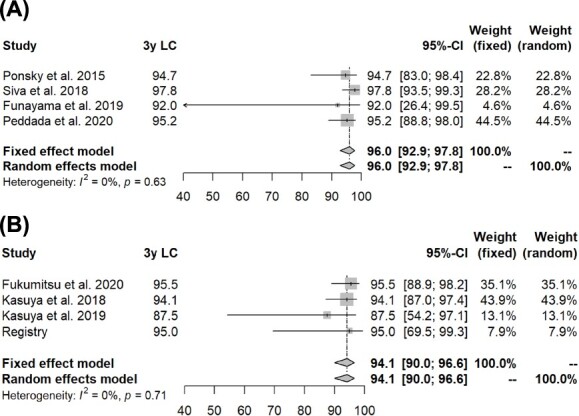
Comparisons of LC rates between SBRT and PBT based on a random effects model. SBRT (A) and PBT (B).

Based on the random effects model, a meta-analysis comparing SBRT and PBT in terms of 3-year OS, PFS and LC rates after treatment was conducted by a statistician (K.M.). Results obtained from the registry data set in the present study were also included in the analysis. Figure 3 shows the 3-year LC rates, as a representative outcome, after SBRT (I^2^ = 0%) and PBT (I^2^ = 0%), and Table 3 summarizes the OS, PFS and LC rates. The difference in the LC rate between SBRT and PBT was not significant. On the other hand, the 3-year OS and PFS rates were better following PBT than SBRT in the meta-analysis (*P* = 0.005 and 0.031, respectively).

**Table 3:** Treatment outcomes in studies of SBRT or PBT for renal cell carcinoma

Author (Year)	Reference number	Follow-up (months)	OS (%)	PFS (%)	LC (%)	Reduction in eGFR (ml/min/1.73 m2)	GI toxicity (≥grade 3)
**SBRT**							
Ponsky (2015)	21	13^*^	72 (3y)	NA	100 (3y)	NA	10.5%
Yamamoto (2016)	22	31.2^*^	NA	NA	NA	NA	0%
Siva (2017)	23	24^*^	92 (2 y)	89 (2 y)	100 (2 y)	−11 (2 y)	3%
Siva (2018)	24	NA	82/71 (2/4y)	77/65 (2/4 y)	98/98 (2/4 y)	−5.5 (3.6 y^**^)	1.3%
Funayama (2019)	25	48.3^*^	92/71(2/3 y)	NA	92/92 (2/3 y)	−16.3 (2 y^**^)	0%
Peddada (2020)	26	78^*^	88 (5 y)	88 (5 y)	100 (5 y)	−6.8	0%
**Proton beam therapy**							
Fukumitsu (2020)	12	37^*^	95 (3 y)	100 (3 y)	95 (3 y)	−7.2 (3 y^**^)	0%
**CIRT**							
Kasuya (2018)	27	79^*^	89 (5 y)	94 (5 y)	69 (5 y)	−6.1	0%
Kasuya (2019)	28	50^*^	88 (3 y)	100 (3 y)	88 (3 y)	−10.8	0%

*Abbreviations*: SBRT, stereotactic body radiotherapy; CIRT, carbon-ion radiotherapy; OS, overall survival; PFS, progression-free survival; LC, local control; eGFR, estimated glomerular filtration rate; GI, gastrointestinal; NA, not assessed

^*^median

^**^estimated

## DISCUSSION

RT may be a non-surgical treatment option for RCC patients with contraindications for surgery, RFA, or cryoablation. However, normal tissues surrounding RCC tumors, including kidney, GI tract and lung tissues, are radiosensitive, whereas RCC is considered to be radioresistant. Therefore, definitive RT has limited usefulness for primary RCC, although photon-based SBRT has been attempted in the last decade [29]. From the present study, we found that scientific publications on primary RCC treated with RT are limited in number. Regarding PBT, only four studies including ours, all conducted by Japanese researchers, were identified [12, 27, 28, 30], of which three were included in the present systematic review.

We previously conducted the first nationwide study involving all proton beam therapy facilities in Japan to confirm the efficacy of hypofractionated proton beam therapy for primary RCC; data from 22 RCC patients treated at six Japanese institutions were analyzed [12]. In that study, the 3-year OS and disease-specific survival rates were 95% and 100%, respectively, and no recurrence occurred over a median follow up of 37 months. Regarding CIRT, the optimal dose fractionation schedule was determined by phase I/II dose escalation studies, and the number of fractions was reduced from 16 over 4 weeks to 12 over 3 weeks; another study of 4-fraction CIRT is being conducted [8]. No severe GI toxicities were observed in any of the CIRT studies. In the present study, evaluation of the feasibility and efficacy of hypofractionated PBT for primary RCC using a multi-institutional registry data set was performed in addition to a systematic review. The 3-year OS and PFS rates after PBT for the 20 patients registered in the cohort for 2 years were 100% and 88.9%, respectively, and no grade ≥ 3 GI toxicity was observed. Overall, the prospective registry data seem to support the clinical outcomes in terms of feasibility and efficacy reported in previous studies.

**Fig. 4:**
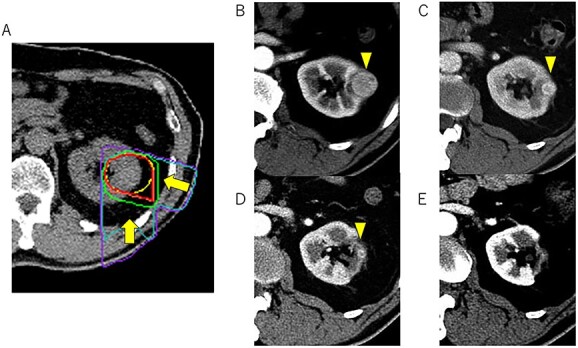
Representative changes in the dose distribution in an irradiated tumor. Dose distribution of carbon-ion radiotherapy (A) before treatment (B) and at 1 year (C), 3 years (D), and 5 years (E) after treatment.

No randomized trials directly comparing SBRT and PBT as curative RT for primary RCC were identified in the systematic review. Therefore, a meta-analysis of retrospective studies was performed based on a random effects model. Although the proportion of non-T1 tumors was higher in the PBT studies than SBRT studies (weighted average: 14.2% vs 2.3%), the LC rate after PBT was similar to that after SBRT (94.1% after PBT vs 96% after SBRT). Generally, the radiation dose is closely correlated with the ability to control radioresistant tumors. RCC cells are usually slow growing and exhibit a lower α/β ratio based on the linear quadratic model, but their growth exponentially decreases at single radiation doses exceeding 6 Gy [18]. Thus, dose escalation with hypofractionation using a high fractional dose is a reasonable approach to improve the therapeutic ratio of RT in RCC patients as well as prostate cancer patients [31]. In reality, in a systematic review, Correa *et al.* [11] reported a high LC rate after SBRT (97.2%, 95% CI: 93.9–99.5), and the local failure rate was higher in the low-dose than high-dose SBRT arm. Since no obvious difference in the BED_5_ between SBRT and PBT was observed in the present study (Table 2), the finding of similar LC rates between the treatments was reasonable. Therefore, the systematic review confirmed that SBRT and PBT using a hypofractionation regimen are very effective treatments for primary RCC. Taking into consideration these favorable tumor control rates after hypofractionated RT, the radiosensitivity of RCC should be reconsidered. Funayama *et al.* [25] and Nomiya *et al.* [30] reported that in Japanese patients, RCC tumors were gradually reduced in size after SBRT and CIRT and sometimes transiently progressed. In our experience, the tumors also continuously shrunk for more than several years after CIRT (Fig. 4). Considering these features, we should pay attention to the RCC tumor response, as RCC may not be radioresistant but rather a slow responder to RT.

Because RCC tumors are sometimes close to the GI tract, including the stomach, duodenum and colon, GI toxicities is of concern in SBRT for them [21, 23, 24]. On the other hand, no severe GI toxicities were consistently reported in the PBT series including the present registry, in which 75% of the tumors located at the inner/ventral areas of the kidney. Although the effectiveness and feasibility of PBT cannot be directly compared with those of SBRT in the RCC treatment, the physical properties of charged particles might cause less GI toxicity. On the other hand, there was no obvious difference between SBRT and PBT in eGFR reduction, a benchmark of renal function. However, the radiation-induced vascular damage in the normal kidney is considered a late effect of RT, and the eGFR was also continually reduced after RT. Siva *et al.* [32] reported an apparent dose–response relationship in renal function after SBRT, with a plateau exceeding 100 Gy when normalized to the BED_3_. Theoretically, the larger the tumor size, the greater the kidney volume irradiated by low-to-intermediate SBRT doses. On the other hand, Kasuya *et al.* [27] showed minimal loss of renal function after CIRT in patients without renal comorbidities pretreatment. Since charged particles can be limited to inside the edge of the target, as shown in Fig. 4, changes in the volumes irradiated by low-to-intermediate doses are minimal with PBT [33]. Furthermore, the present systematic review indicated that approximately 10% of patients treated with PBT had large-sized tumors (non-T1). Taken together, PBT appears to be a safe treatment, especially for large tumors.

No randomized trials have compared PBT with SBRT in primary RCC patients. Therefore, we performed not only a systematic review but also a meta-analysis to clearly determine the effectiveness of PBT on RCC. Although the survival outcomes were better after PBT than after SBRT, the data were still unreliable because their comparisons were performed without any adjustments. As long-term follow-up data and much effort are necessary to conduct randomized trials, comparisons using other techniques such as a model-based approach would be reasonable [34]. There are additional limitations in the present study. First, the number of patients in each study was small, and three of nine studies listed in the present systematic review were retrospective. Second, the characteristics of the patients and treatments such as the dose fractionation schedules and definitions of the target volumes were heterogenous. Since 2016, PBT for all RCC patients under advanced medical treatment in Japan has been performed in accordance with the treatment policy of JASTRO, and all data were collected prospectively. Therefore, we will be able to obtain more reliable and detailed data in the near future.

According to discussions with the systematic review team and two expert evaluators (W.O. and H.A.) from the Japanese Urological Association, our present study confirmed that prospectively collected registry data reproduced outcomes of previously published PBT studies for RCC. Furthermore, PBT is expected to result in similar, if not superior, treatment results compared with photon-based SBRT for primary renal cancer. In particular, PBT is effective even for large RCC and provides a therapeutic option when photon-based SBRT is not indicated. Further accumulation of detailed data over long-term follow up will validate the efficacy of PBT.

## CONFLICT OF INTEREST

The authors confirm they have no conflicts of interest to declare.

## DATA AVAILABILITY

The data underlying this article will be shared on reasonable request to the corresponding author.
